# Drug conjugation to hyaluronan widens therapeutic indications for ovarian cancer

**DOI:** 10.18632/oncoscience.150

**Published:** 2015-03-23

**Authors:** Isabella Monia Montagner, Anna Merlo, Debora Carpanese, Gaia Zuccolotto, Davide Renier, Monica Campisi, Gianfranco Pasut, Paola Zanovello, Antonio Rosato

**Affiliations:** ^1^ Veneto Institute of Oncology IOV - IRCCS, Padua, Italy; ^2^ Department of Surgery, Oncology and Gastroenterology, University of Padua, Padua, Italy; ^3^ Department of Medicine, University of Padua, Padua, Italy; ^4^ Fidia Farmaceutici S.p.A, Abano Terme, Italy; ^5^ Department of Pharmaceutical and Pharmacological Sciences, University of Padua, Padua, Italy

**Keywords:** ovarian cancer, tumor targeting, SN-38, hyaluronan, bioconjugate

## Abstract

Management of ovarian cancer still requires improvements in therapeutic options. A drug delivery strategy was tested that allows specific targeting of tumor cells in combination with a controlled release of a cytotoxic molecule. To this aim, the efficacy of a loco-regional intraperitoneal treatment with a bioconjugate (ONCOFID-S) derived by chemical linking of SN-38, the active metabolite of irinotecan (CPT-11), to hyaluronan was assessed in a mouse model of ovarian carcinomatosis.

*In vitro*, the bioconjugate selectively interacted with ovarian cancer cells through the CD44 receptor, disclosed a dose-dependent tumor growth inhibition efficacy comparable to that of free SN-38 drug, and inhibited Topoisomerase I function leading to apoptosis by a mechanism involving caspase-3 and -7 activation and PARP cleavage. *In vivo*, the intraperitoneal administration of ONCOFID-S in tumor-bearing mice did not induce inflammation, and evidenced an improved therapeutic efficacy compared with CPT-11.

In conclusion, SN-38 conjugation to hyaluronan significantly improved the profile of *in vivo* tolerability and widened the field of application of irinotecan. Therefore, this approach can be envisaged as a promising therapeutic strategy for loco-regional treatment of ovarian cancer.

## INTRODUCTION

Ovarian cancer (OC) represents a serious clinical entity, with estimated 21,980 new cases and 14,270 deaths in U.S. in 2014 [1]. The vast majority of patients with epithelial OC tends to present with advanced stage disease, when it has already metastasized throughout the peritoneal cavity and to the upper abdomen, resulting in a low overall cure rate and high percentage of chemoresistance [2]. Hence, new therapeutic approaches are imperatively needed, in particular an efficient drug delivery strategy capable of targeting highly disseminated and aggressive tumor cells. This task can be accomplished by exploiting cancer-specific receptors and the related ligands, CD44 and hyaluronic acid (HA) representing a notable example.

HA is a linear polysaccharide naturally present in the extracellular matrix, the synovial fluid of joints and the cartilage and hence fully biocompatible [3, 4]. HA is the primary ligand for CD44, that is overexpressed in many cancer types including the ovarian histotype [5]. Therefore, the conjugation of a drug to hyaluronan can confer specificity and selectivity for cancerous cells, and can also provide a pharmacological advantage in terms of solubilization and stabilization. This feature is of inestimable value for those drugs whose high hydrophobicity precludes the clinical use, as in the case of the active metabolite SN-38 of the pro-drug irinotecan (CPT-11), which is about 100-fold more potent than its precursor [6]. The direct administration of SN-38 could overcome the limitations due to the irinotecan metabolism that is quite inefficient (approximately only 10% of irinotecan is converted to SN-38) and characterized by a considerable patient-to-patient variability. Interestingly, while irinotecan is usually employed in colon cancer, it has been recently used with success also against ovarian cancer, in particular in those cases refractory to paclitaxel and carboplatin, the first-line post-operative treatment option [7-9].

Several approaches are currently under investigation to allow SN-38 administration, among which inclusion into nanoparticles [10], liposomes [11] and micelles [12], and conjugation with polyamidoamine dendrimers [13] or carbohydrates [2].

Here, we report that a bioconjugate based on the chemical linking of SN-38 to HA (ONCOFID-S) retains a comparable activity respect to the free drug *in vitro*, but performs better *in vivo* in OC xenograft mouse models compared to the CPT-11 pro-drug. Moreover, the bioconjugate significantly improves the tolerability profile of the local treatment. These results make ONCOFID-S a promising drug for the loco-regional treatment of ovarian cancer, thus supporting its testing in a clinical setting.

## RESULTS

### Evaluation of HA receptor expression

CD44 and RHAMM are considered the main receptors for hyaluronan binding. The expression of these receptors in IGROV-1, OVCAR-3 and SKOV-3 tumor cell lines was analyzed by flow cytometry. CD44 was intensely expressed in all cell lines tested, whereas RHAMM was mildly present only at intracellular level with the exception of OVCAR-3 that exhibited also a weak extracellular expression (Fig. S1).

### Analysis of interaction of ONCOFID-S with target cancer cell lines

**Figure 1:**
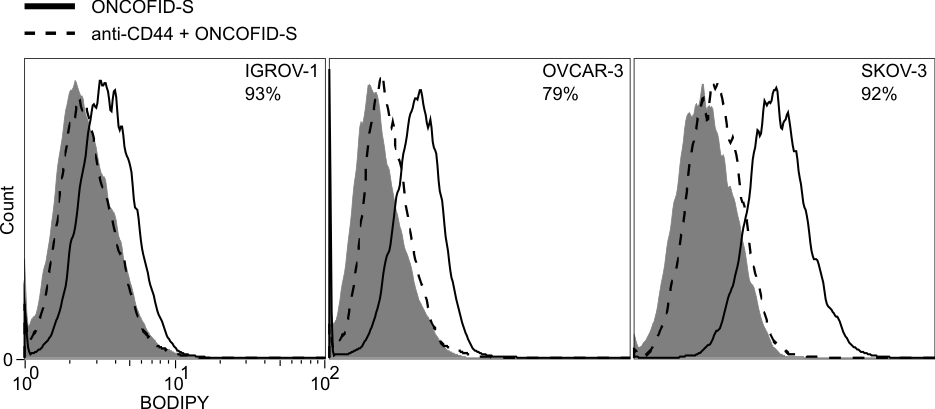
Blocking of the bioconjugate/receptor interaction by an anti-CD44 antibody IGROV-1, OVCAR-3 and SKOV-3 tumor cell lines were incubated with BODIPY-labeled ONCOFID-S alone (solid line) or in the presence of an anti-CD44 blocking mAb (dashed line), and analyzed by flow cytometry. Grey plot depicts isotype control. Data at the upper-right corner of each panel report the percentage of reduction induced by anti-CD44 mAb blocking treatment.

The role played by CD44 in the interaction between cells and the bioconjugate was assessed in a competition assay where target cancer cell lines were incubated with an anti-CD44 blocking mAb, and then with BODIPY-labeled ONCOFID-S. The blocking antibody induced a shift in the cell-bound fluorescent signal as compared to the cells incubated with the bioconjugate only (Fig. 1), thus demonstrating the pivotal role of CD44 in the interaction of the hyaluronan-based bioconjugate with tumor cells.

### ONCOFID-S inhibits Topo I activity and induces apoptosis

**Figure 2:**
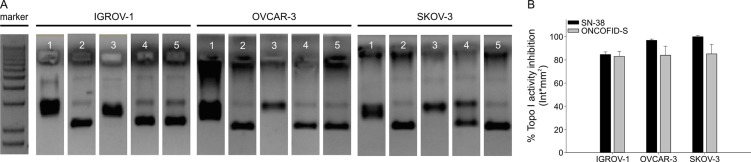
Assessment of bioconjugate mechanism of action (A) Inhibition of Topoisomerase I activity after ONCOFID-S or SN-38 treatment in IGROV-1, OVCAR-3 and SKOV-3 cell lines. Gels show the supercoiled or relaxed forms of pBR322 plasmid after incubation with a 1:100 dilution of nuclear protein neat extracts obtained from tumor cells treated with conjugated or free drug for 4 hours. Marker; lane 1, relaxed pBR322 plasmid (positive control); lane 2, supercoiled plasmid (negative control); lane 3, supercoiled plasmid in the presence of nuclear protein neat extract from drug-untreated cells; lane 4, supercoiled pBR322 admixed with nuclear protein neat extract from ONCOFID-S-treated cells; lane 5, supercoiled pBR322 admixed with nuclear protein neat extract from SN-38-treated cells. (B) The quantification of the reaction shown in A. Figure reports mean ± SD of 3 independent experiments.

SN-38 is the active metabolite of irinotecan and interacts specifically with the enzyme Topo I, blocking its property to relieve torsional strain in DNA by inducing reversible single-strand breaks. To evaluate the effect of ONCOFID-S and SN-38 on Topo I activity, nuclear protein extracts from treated cells were incubated with a supercoiled plasmid DNA (pBR322) and the ratio between the supercoiled and relaxed forms was visualized (Fig. 2A) and quantified (Fig. 2B) by gel electrophoresis. In all tumor cell lines, both the bioconjugate and the free drug presented the same inhibitory activity towards Topo I, thus indicating that the active SN-38 molecules are released by ONCOFID-S and have access to the nucleus where they can block the enzyme activity.

**Figure 3:**
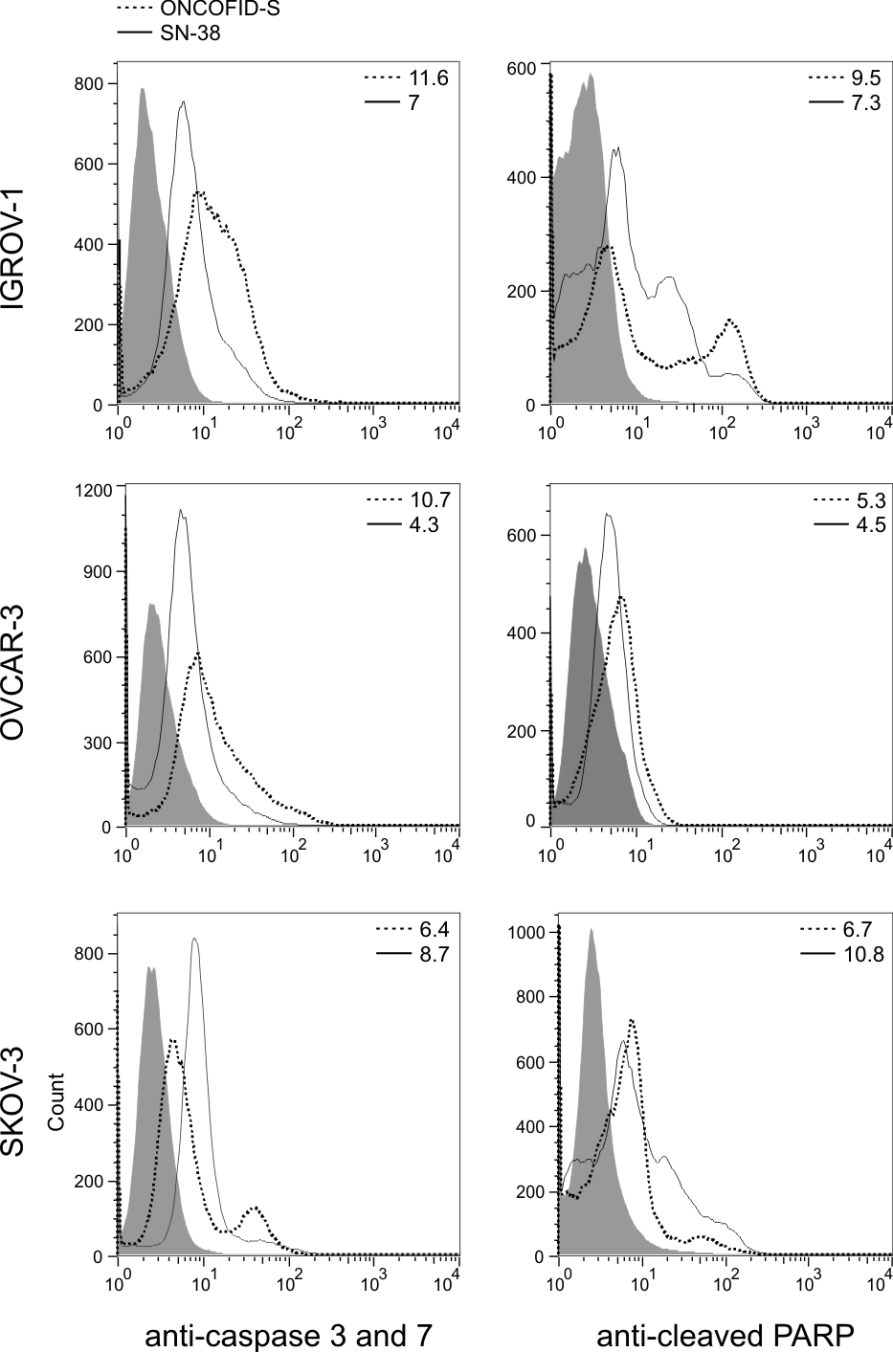
Apoptosis induction by free and conjugated drug (A) IGROV-1, OVCAR-3 and SKOV-3 cell lines were incubated with ONCOFID-S (dotted line) or free SN-38 (solid line) for 6 hours and stained with a fluorescent probe detecting active caspase 3 and 7. (B) After a 12-hour treatment with ONCOFID-S (dotted line) or free SN-38 (solid line), the same ovarian cancer cell lines were labeled with a FITC-conjugated anti-cleaved PARP mAb. Grey plot depicts isotype control.

Irinotecan has been previously reported to be capable of activating apoptosis [14]. To assess the capacity of the bioconjugate to induce apoptosis as well, the activation of caspase 3 and 7 and the cleavage of PARP were evaluated on target cells. Tumor cell lines were incubated with ONCOFID-S or SN-38, and stained with a fluorochrome-labeled probe that binds covalently to the active caspase 3 and 7 or a FITC-labeled anti-human cleaved PARP mAb. Cytometry analysis disclosed that both treatments induced similar activation of caspase cascade and similar levels of cleaved PARP (Fig. 3), leading to overlapping effects on apoptosis induction.

### Tumor cell growth inhibitory activity of ONCOFID-S

To test ONCOFID-S activity, IGROV-1, OVCAR-3 and SKOV-3 target cell lines were incubated with escalating concentrations of the bioconjugate and the dose-dependent growth inhibitory activity was compared to that of the free irinotecan metabolite. IC_50_ of ONCOFID-S was comparable to that observed for SN-38 (Table S1). No toxic effect could be ascribed to hyaluronan (data not shown).

### Effects on the peritoneal lining

**Figure 4:**
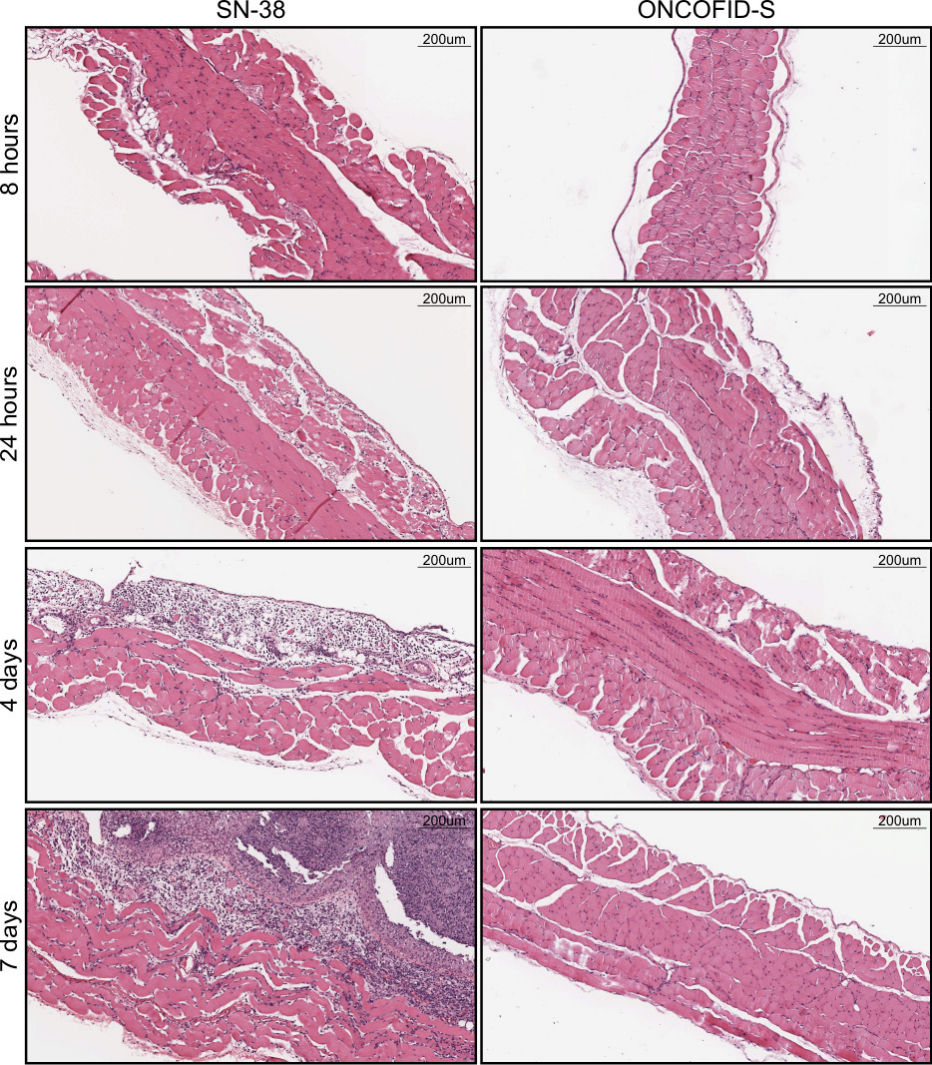
Local tolerability study Histological analysis were carried out on peritoneal mesothelial lining at different time points (8 hours, 24 hours, 4 days and 7 days) after i.p. injection of SN-38 or ONCOFID-S. (H&E staining; original magnification, x10).

In a clinical setting, locally administered antitumor therapy must be well tolerated. To assess whether ONCOFID-S induced irritating effects on the peritoneal mesothelial lining, three groups of BALB/c mice were injected once i.p. with the bioconjugate, CPT-11 or SN-38. Samples of the abdominal wall were then collected at different time points after treatment and analyzed histologically (Fig. 4 and Fig. S2). In particular, in ONCOFID-S-treated mice, mesothelium did not show inflammatory infiltrates or morphological alterations at any time. Conversely, SN-38 brought about a strong inflammatory reaction at both the mesothelial lining and the muscle abdominal wall, which was already evident after 24 hours and became more pronounced the following days.

### ONCOFID-S inhibits OC tumor growth *in vivo* and prolongs survival of treated mice

The *in vivo* antitumor therapeutic activity of ONCOFID-S was evaluated in mice bearing OC xenografts. SCID mice were inoculated i.p. with IGROV-1 or SKOV-3 cell lines, and treated locally with ONCOFID-S or the free CPT-11 through the i.p. route. We administered the precursor irinotecan instead of its active metabolite because of poor water solubility, high side effects and instability of SN-38 at a physiological pH [15], factors that limit its clinical use. The therapeutic impact of the different treatments was evaluated by bioluminescence imaging.

**Figure 5:**
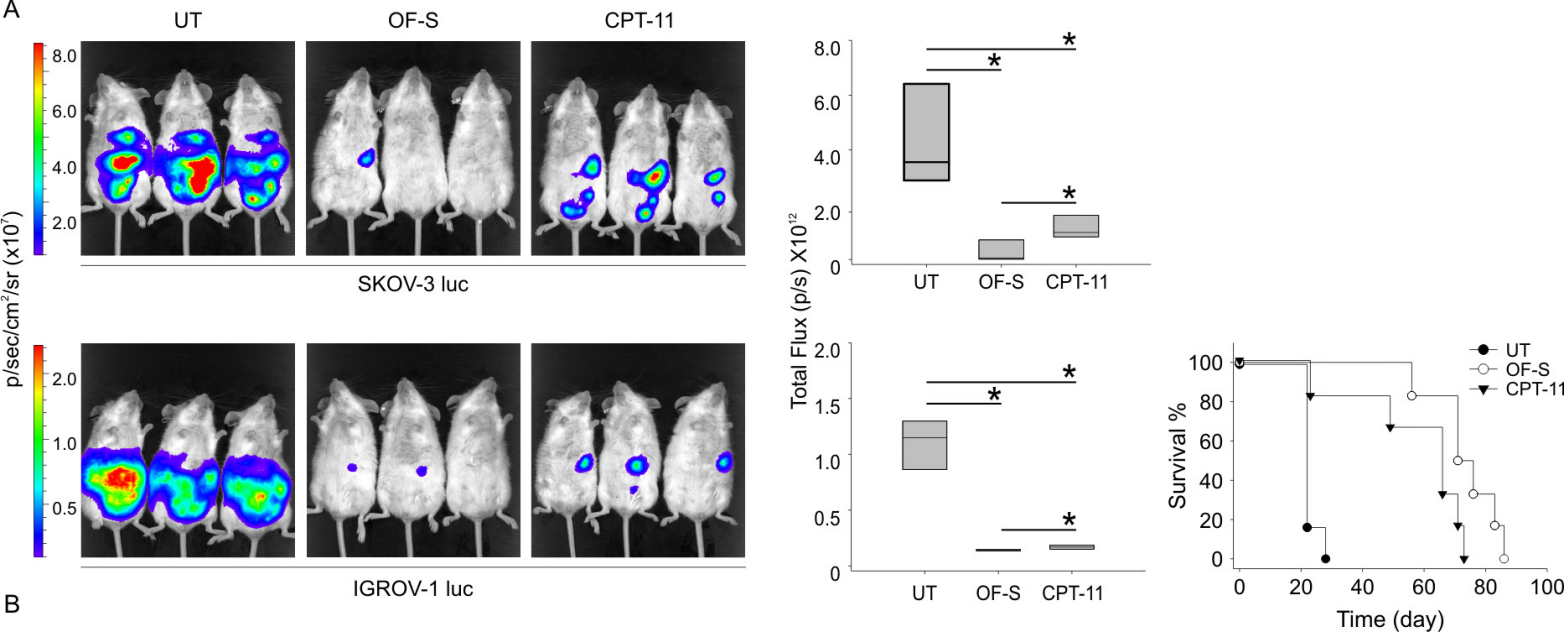
Assessment of in vivo tumor growth and response to therapy Bioluminescence imaging of treated or untreated mice inoculated with 5 × 10^6^ bioluminescent SKOV-3 (A) or IGROV-1 (B) cells. Panels show three representative mice per group at 25 days after tumor injection. The vertical box plot represented the cumulative total photons emission from mice treated or untreated at day 25. Six mice per group were analyzed and data represent means ± SD. Kaplan-Meier survival curves of mice inoculated with IGROV-1 (B) cells and treated with ONCOFID-S (OF-S; open circles), CPT-11 (filled triangle) or untreated (UT; filled circles).

Results of a pilot study carried out with mice engrafted with SKOV-3 cells showed that ONCOFID-S loco-regional treatment had a relevant inhibitory effect on their growth as compared to untreated as well as CPT-11-treated mice (p = 0.03 and p = 0.05, respectively; Fig. 5A). When the bioconjugate was tested in IGROV-1-bearing mice in a confirmatory experiment, ONCOFID-S not only disclosed a strong antitumor activity *vs.* controls (p = 0.004; Fig. 5B), but resulted even more effective than CPT-11 (p = 0.05).

These results were confirmed by survival analysis in IGROV-1-bearing mice. Indeed, ONCOFID-S treated animals (median survival, 71 days) underwent a strong therapeutic effect and presented an increased survival compared to both untreated control mice (median survival, 22 days; p = 0.001) and CPT-11-treated animals (median survival, 66 days; p = 0.004; Fig. 5B, right panel).

## DISCUSSION

Irinotecan is a synthetic derivative of camptotecin and a FDA-approved drug for the treatment of colorectal carcinoma. It has been also showed to potentially represent a promising drug for ovarian cancer, in both preclinical [16] and clinical studies [17]. In particular, it has been advanced in cases of platinum- and taxane-resistant and refractory disease, a clinical setting where chemotherapy options are very limited. However, it should be noted that only few papers relate to the use of irinotecan as a single agent [17-19], while the majority reports the association of irinotecan with other drugs, such as docetaxel [9], etoposide [20] and cisplatin [21, 22], thus making difficult to discriminate the contribution of each drug to the outcome described. Noteworthy, all these clinical studies involved the administration of the drug by the i.v. route, with significant (up to grade 3 and 4) adverse events such as leukopenia, thrombocytopenia and diarrhea [23]. A loco-regional administration of irinotecan is quite uncommon, despite the fact that the carboxylesterase enzymes responsible for the conversion of the pro-drug to the active SN-38 metabolite are present also in the peritoneum [24, 25]. This is of particular interest in the case of ovarian cancer; in fact, due to the lack of reliable screening tests and to the non-specificity of signs and symptoms, the vast majority of epithelial ovarian cancer patients presents at a diagnosis with an advanced stage disease, mainly involving the peritoneum. This condition, usually seen as dismal, could potentially benefit of a highly aggressive loco-regional therapy. Indeed, the peritoneum-plasma barrier represents a drawback for pharmacological treatments given i.v., but on the other hand can sustain a local delivery of even high concentrations of drugs, thus reducing and sparing heavy systemic side effects. In this regard, the association of cytoreductive surgery (CS) with hyperthermic intraperitoneal chemotherapy (HIPEC) has proved to be effective in improving survival [26].

Here, we presented a drug delivery system involving the use of a HA-based bioconjugate that has the potentiality to improve the loco-regional treatment of OC, in particular in terms of safety and tolerability. Despite clinical successes in fact, CS plus HIPEC remains a great challenge for both clinicians (a particular technical expertise is required) and patients (great morbidity, complications such as intraperitoneal adhesions, infections etc.), so that only patients with good performance status can be candidates for this procedure [26].

We previously showed that a paclitaxel-HA conjugate exerted a relevant therapeutic activity against OC in a preclinical setting [27], and was also very efficient towards bladder cancer both in experimental [28, 29] and clinical conditions [30]; moreover, ONCOFID-S has been already successfully tested in a mouse model of colorectal peritoneal carcinomatosis [6], thus prompting us to assess its potential therapeutic use for OC.

In addition to its well-known biocompatibility, hyaluronan can act as a suitable carrier being endowed with the capability to selectively target tumor cells through the binding to CD44. This receptor is over-expressed in many cancers, including the ovarian histotype. Indeed, such selectivity of ONCOFID-S was confirmed by competition studies with an anti-CD44 blocking mAb. Importantly, the chemical linking of SN-38 to HA did not impact the activity of the drug, as demonstrated by the ability of ONCOFID-S to inhibit *in vitro* Topo I function and to induce apoptosis in ovarian cancer cells, effects that were similar to those exerted by the free molecule. In turn, these features present an important practical follow-out on the anti-tumor efficacy *in vitro* and also *in vivo*, both shortly after the drug administration schedule has been completed when assessing tumor growth by imaging, and long-term when considering survival. In fact, the bioconjugate significantly reduced the short-term tumor burden in both SKOV-3- and IGROV-1-engrafted mice, performing even better than CPT-11. Moreover, ONCOFID-S significantly prolonged survival of IGROV-1-induced peritoneal carcinomatosis respect to the related free drug.

Apart such therapeutic successes, the conjugation with HA brings about additional relevant advantages. From a pharmacological point of view, it drastically increases the water solubility of SN-38 thus allowing its direct administration. In this regard in fact, SN-38 is at least 100-fold more active *in vitro* than CPT-11 at equimolar concentrations, but its clinical use is precluded because of the intrinsic toxicity and extremely low water solubility [6]. Moreover, it has been shown that HA has *per se* the capacity to reduce postoperative and disease-related adhesions without impacting the metastatic potential of tumor cells [31]. From a clinical point of view, the therapeutic outcome can be achieved with negligible adverse effects. Indeed, histological analysis clearly disclosed that the bioconjugate was completely devoid of local toxicity, in line with previously reported data [6, 27], while free SN-38 was strongly inflammatory. In addition, ONCOFID-S has been reported not to cause myelotoxicity, thus potentially representing an attractive candidate for the treatment of UGTA1 genotype patients, who are prone to develop severe neutropenia related to CPT-11 [6].

Overall, present data envisage that the conjugation with HA can provide a strategy to potentially improve the loco-regional treatment of OC, and to widen the use of existing drugs over their formal approval, thus increasing the portfolio of potential therapeutic options.

## MATERIALS AND METHODS

### Drugs

The hyaluronan-SN-38 bioconjugate, (ONCOFID-S, Fidia Farmaceutici, Abano Terme, Italy), has been previously described [6]. The batch of ONCOFID-S used was characterized by a SN-38 loading of 9.4%. Irinotecan (CPT-11) and SN-38 were purchased from Antibioticos (Rodano, Italy). When needed, bioconjugate was labeled with the fluorochrome BODIPY TR cadaverine (Invitrogen, San Giuliano Milanese, Italy), as previously described [27].

### Tumor cell lines

The following human ovarian cancer cell lines were used: IGROV-1, OVCAR-3 and SKOV-3. Cells were grown in RPMI 1640 (EuroClone, Milan, Italy) supplemented with 10% (v/v) heat-inactivated fetal bovine serum (Gibco BRL, Paisley, UK), 2 mM L-glutamine (Gibco BRL), 10 mM HEPES (PAA Laboratories, Linz, Austria), 200 U/mL penicillin (Pharmacia & Upjohn, Milan, Italy), 200 U/mL streptomycin (Bristol-Mayers Squibb Italia, Rome, Italy) and 1 mM sodium pyruvate (Lonza, Basel, Switzerland), hereafter referred as to complete medium. Cell lines were maintained at 37°C in a humidified atmosphere containing 5% CO_2_.

### Viability assay

The determination of *in vitro* drug cytotoxicity was assessed by the ATPlite luminescence adenosine triphosphate (ATP) detection assay system (PerkinElmer, Zaventem, Belgium), according to the manufacturer's instructions. All ovarian cancer cell lines were resuspended in complete medium and seeded into 96-well flat-bottomed plates (8 × 10^3^/well). The day after, different concentrations of drugs were added (final volume, 100 μL/well) for 72 hours. At day 4, 50 μL of lysis solution were added to each well followed by addition of 50 μL of substrate solution. Finally, the luminescence was counted by the TopCount Microplate Counter (PerkinElmer). Within each experiment, determinations were performed in triplicate and experiments were repeated 5 times for each cell line. The percentage of cell survival was calculated by determining the counts per second (cps) values according to the formula: [(cps_tested_ − cps_blank_) / (cps_untreated control_ − cps _blank_)] × with 100, with cps_blank_ referring to the cps of wells that contained only medium and ATPlite solution. The IC_50_ values were calculated from semi-logarithmic dose-response curves by linear interpolation.

### Flow cytometry analysis

CD44 and CD168 (RHAMM) expression in all tumor cell lines was evaluated by flow cytometry, as previously detailed [27]. Direct interaction of ONCOFID-S with cancer cell lines was assessed by incubating cells with BODIPY-labeled bioconjugate (6 μg/ml in SN-38 equivalent) for 30 minutes in the presence of an anti-CD44 blocking mAb (10 μg/ml, clone 5F12, Lifespan Biosciences, Seattle, WA). Apoptosis induction was evaluated in terms of activation of the caspases 3 and 7, as previously reported [29], and by determining the percentage of the cleaved form of poly ADP-ribose polymerase (PARP). In this case, ovarian cancer cell lines were incubated with free SN-38 (50 μg/mL) or ONCOFID-S (50 μg/ml in SN-38 equivalent) for 12 hours at 37°C, washed twice, and labeled with FITC-conjugated anti-cleaved PARP mAb (Becton Dickinson, Franklin Lakes, NJ). All experiments were carried out using a flow cytometer FACS-Calibur (Becton Dickinson) and the FlowJo 7.6.5 data analysis software package (TreeStar, USA).

### Assessment of Topoisomerase I activity by plasmid DNA relaxation assay

Topoisomerase I (Topo I) was isolated from tumor cell lines by Qproteome Nuclear Protein Kit (Qiagen, Milan, Italy), after incubation of cells (5 × 10^6^/sample) with ONCOFID-S (50 μg/mL in SN-38 equivalents), SN-38 (50 μg/mL) or complete medium (untreated cells) at 37°C for 1 hour. Enzyme activity was assessed using the Human Topo I Assay Kit for cell extracts (Inspiralis, Norwich, United Kingdom). Dilutions of cell extracts (1:5, 1:10, 1:50, 1:100 and 1:500) were incubated for 30 minutes at 37°C with the relaxation mix containing a supercoiled DNA substrate (pBR322). Reaction was stopped by adding an equal volume of chloroform/isoamyl alcohol (24:1). Samples were fractionated by 0.8% agarose gel electrophoresis, visualized by ethidium bromide staining and quantified by UV densitometry using the supercoiled and relaxed pBR322 plasmid as positive or negative control, respectively. Inhibition of Topo I activity was calculated as the ratio between the supercoiled fractions in treated cells and the positive control and expressed as percentage.

### Mice

Six to eight week-old female severe combined immunodeficiency (SCID) and BALB/c mice were purchased from Charles River Laboratories (Calco, Italy), and housed in our Specific Pathogen Free (SPF) animal facility. Procedures involving animals and their care were in conformity with institutional guidelines (D.L. 116/92 and subsequent implementing circulars), and experimental protocols (project ID: 3/2012) were approved by the local Ethical Committee of Padua University (CEASA). During *in vivo* experiments, animals in all experimental groups were examined daily for a decrease in physical activity and other signs of disease or drug toxicity; severely ill animals were euthanized by carbon dioxide overdose.

### Local tolerability analysis

To assess the potential irritating effect of bioconjugate and free drug on peritoneal mesothelium, BALB/c mice were injected once i.p. with 200 mg/kg (19.2 mg/kg in SN-38 equivalents) of ONCOFID-S, 60 mg/kg of CPT-11 or 19.2 mg/kg of SN-38 (8 mice/group). At different time points thereafter (8 hours, 24 hours, 4 days and 7 days), two animals per time point were sacrificed and fragments of their abdominal wall were collected for morphological analysis. For histologic evaluation, tissue samples were fixed in 4% neutral-buffered formalin, embedded in paraffin, sectioned at 4 μm and stained with hematoxylin and eosin.

### Anti-tumor activity *in vivo* and optical imaging

SCID mice were inoculated i.p. with 5 × 10^6^ IGROV-1 or SKOV-3 tumor cells. Pharmacological treatments were started at day 7 from cancer cell lines injection and carried out according to a q7dx3 schedule (every 7 days for 3 doses). Each experiment comprised groups of animals (six mice/group) that received ONCOFID-S i.p. (200 mg/kg, 19.2 mg/kg in SN-38 equivalents), CPT-11 i.p. (60 mg/kg), or were left untreated. Injected tumor cells had been previously transduced with a lentiviral vector coding for the firefly luciferase reporter gene [32] to track tumor growth *in vivo*. Bioluminescence (BLI) images were acquired at different time points after *in vivo* cell injection using the IVIS Lumina II Imaging System (PerkinElmer). Ten minutes before each imaging session, animals were anesthetized with isoflurane/oxygen and administered i.p. with 150 mg/kg of D-luciferin (PerkinElmer) in Dulbecco's Phosphate-Buffered Saline (DPBS). A constant region of interest (ROI) was manually selected around the abdomen of animals and the signal intensity was measured as radiance (photon/sec) using the LivingImage software 3.2 (PerkinElmer). Tumor growth and response to therapy were monitored by BLI and by recording survival.

### Statistical analysis

Survival curves and probabilities were estimated using the Kaplan-Meier technique. A log-rank test for comparisons or an Anova test were used when required. Analysis of data were done using the MedCalc (version 12) and SigmaPlot (version 12.3) statistical packages.

## SUPPLEMENTARY MATERIAL FIGURES AND TABLE


