# Supplementary material for: Drug conjugation to hyaluronan widens therapeutic indications for ovarian cancer
Source: Oncoscience. 2015 Mar 23;2(4):373–81. doi: 10.18632/oncoscience.150 (PMC4468323; doi:10.18632/oncoscience.150)
Supplement: Supplementary file 1 [file oncoscience-02-0373-s001.pdf]

Drug conjugation to hyaluronan widens therapeutic indications for ovarian cancer

Supplemental Material

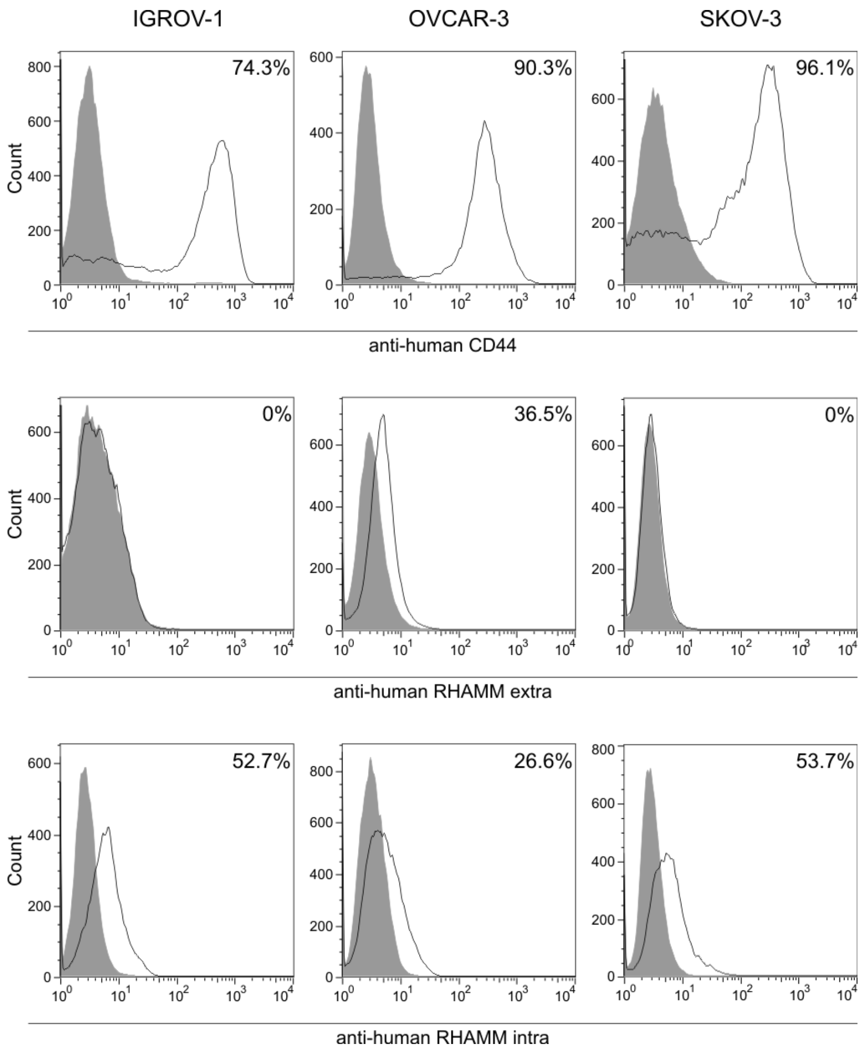

Fig. S1: CD44 and RHAMM expression in human ovarian cancer cell lines. IGROV-1, OVCAR-3 and SKOV-3 viable cells were stained with a FITC-labeled anti-human-CD44 mouse mAb (upper panels). Viable or fixed and permeabilized cells were stained with an anti-human-CD168 mouse mAb followed by an Alexa546-conjugated anti-Ig mouse serum (central and lower panels) to evaluate the surface or intracellular expression of RHAMM, respectively. Grey plot depicts isotype control. Percentage of CD44 and RHAMM expression are reported at the upper right corner of each box.

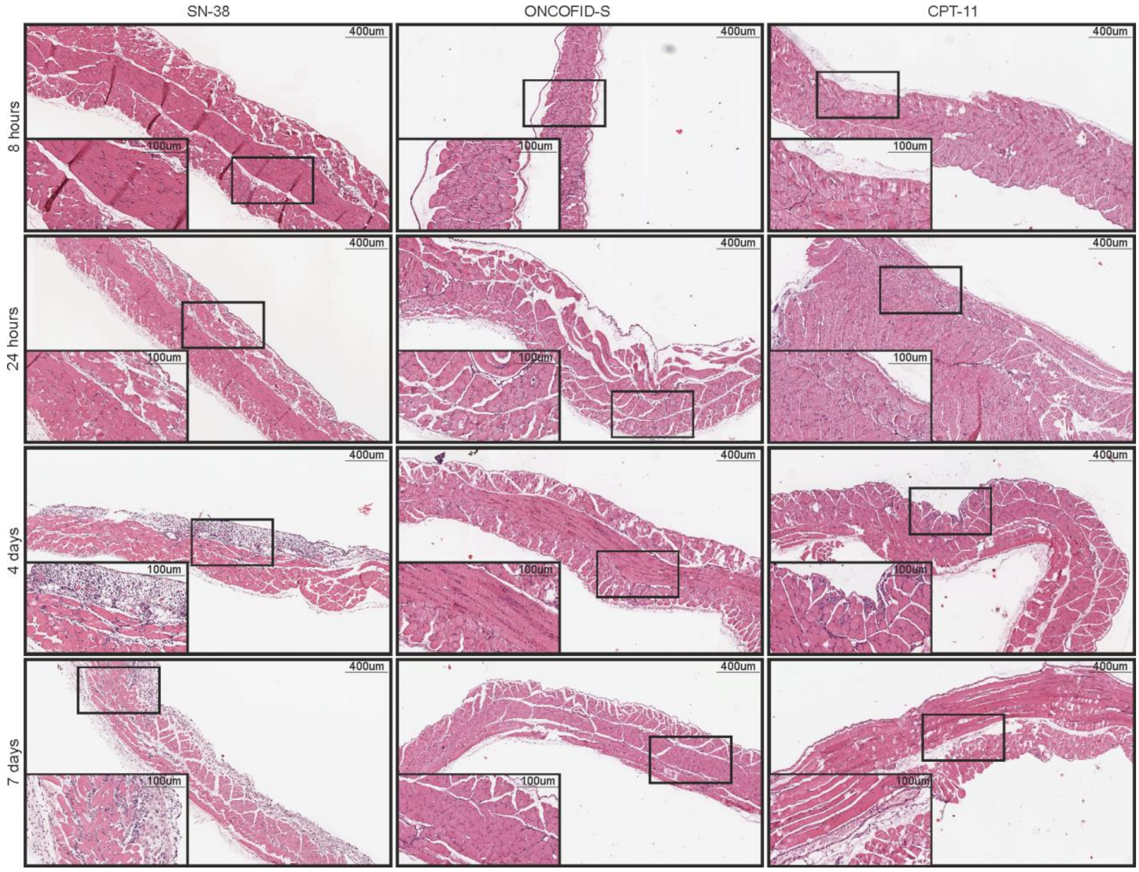

Fig. S2: Local tolerability study. Histological analysis were carried out on peritoneal mesothelial lining at different time points (8 hours and 1, 4 and 7 days) after i.p. injection of ONCOFID-S, CPT-11 or SN-38. (H&E staining; original magnification, x5). Insets, a more detailed view of representative areas (magnification, x20).

IC_{50} in ng/mL ± S.E.		
	OF-S	SN-38
IGROV-1	52 ± 14	42 ± 15
OVCAR-3	37 ± 25	22 ± 18
SKOV-3	62 ±30	9 ± 8

Table S1: IC_{50} values of SN-38 and ONCOFID-S. The values of IC_{50} reported are the mean ± SE of five viability experiments carried out for each tumor cell line. For each experiment, the IC_{50} was calculated from each single semi-logarithmic dose-response curve by linear interpolation, and obtained values were then averaged. Values are reported in ng/mL and for the bioconjugate they are expressed in terms of free drug equivalents.
